# Warfarin-induced impairment of bone material quality in a patient undergoing maintenance hemodialysis: A case report

**DOI:** 10.1097/MD.0000000000020724

**Published:** 2020-06-19

**Authors:** Hiroki Ishii, Satoshi Kurihara, Keiji Hirai, Katsunori Yanai, Susumu Ookawara, Yoshiyuki Morishita

**Affiliations:** a Division of Nephrology, First Department of Integrated Medicine, Saitama Medical Center, Jichi Medical University; b Saitama Tsukinomori Clinic, Saitama, Japan.

**Keywords:** bone material quality impairment, dialysis, histomorphometric analysis, warfarin

## Abstract

**Introduction:**

The use of warfarin in patients undergoing hemodialysis is associated with decreased bone mineral density and an increased incidence of bone fracture. However, no studies to date have directly estimated bone quality with bone histomorphometry in patients with bone abnormalities who are taking warfarin and undergoing hemodialysis.

**Patient concerns:**

A 47-year-old female with Noonan syndrome presented with progressive bilateral lower extremity pain on walking, and skin sclerosis. She had been undergoing maintenance hemodialysis for 25 years following 2 years of peritoneal dialysis for chronic glomerulonephritis. She had been taking warfarin as an anticoagulant agent for 13 years after she underwent an aortic valve replacement.

**Diagnosis:**

Warfarin-induced impairment of bone material quality.

**Interventions and outcomes:**

Histomorphometric analysis of the bone biopsy specimens showed impairment in bone calcification processes, a high turnover of bone remodeling, low bone volume, and mild fibrosis. The bone abnormality could not be categorized into any type of representative bone disease classification such as osteitis fibrosa, osteomalacia, adynamic bone disease, uremic osteodystrophy, or hyperparathyroidism, but was consistent with warfarin-induced impairment of bone material quality.

**Conclusion:**

Warfarin can induce impairment of bone material quality in a patient undergoing hemodialysis.

## 1 Introduction

Patients undergoing hemodialysis have a higher risk of bone fracture due to impairment of bone material quality.^[1]^ Several factors in this population contribute to impairment of bone material quality, including uremic osteodystrophy and secondary hyperparathyroidism.^[2]^ Additionally, several drugs including glucocorticoids, loop diuretics, and warfarin have been shown to impair bone material quality.^[3–5]^ Warfarin is an antagonist of vitamin K that can impair bone mineralization by inhibiting γ-carboxylation of osteocalcin, which is a vitamin K-dependent protein.^[6]^ Vitamin K deficiency is frequently observed in patients undergoing hemodialysis.^[7]^ Thus, in these patients, taking warfarin is considered to increase the impairment of bone material quality resulting in an increased risk of bone fracture. Previous studies have reported that the use of warfarin in patients undergoing hemodialysis is associated with decreased bone mineral density and an increased incidence of bone fracture.^[8,9]^ However, no studies to date have directly estimated bone quality with bone histomorphometry in patients with bone abnormalities who are taking warfarin and undergoing hemodialysis. We herein report a case of warfarin-induced impairment of bone material quality that was evaluated by bone histomorphometry in a patient who was undergoing hemodialysis.

## 2 Case

**Figure 1:**
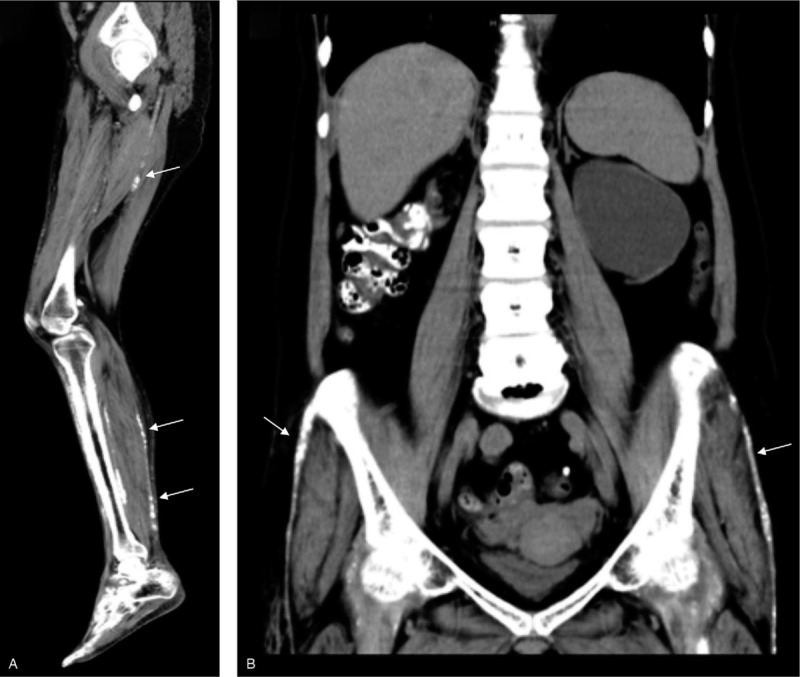
Computed tomography images of lower extremities. The images show calcification of the fascia in the lower extremities (A and B, white arrows).

A 47-year-old female with Noonan syndrome had been undergoing maintenance hemodialysis for 25 years following 2 years of peritoneal dialysis for chronic glomerulonephritis. She had been taking warfarin as an anticoagulant agent for 13 years after she underwent an aortic valve replacement. Her warfarin dose was 2 to 3 mg/d with a prothrombin time-international normalized ratio range from 1 to 2. For 3 years before admission, she experienced pain on walking, skin sclerosis, and a restricted range of joint motion in the bilateral lower extremities, and these symptoms were gradually progressing. Computed tomography showed extensive fascia and tendon calcification of the bilateral lower extremities (Fig. 1A and 1B, white arrows). Additionally, dual-energy X-ray absorptiometry showed low bone mineral density at -4.5 standard deviations compared with the standard value of bone mineral density of a younger age group (20–29 years old) in her radial bone. The patient's chronic kidney disease–mineral and bone disorder (CKD-MBD) was well controlled by taking calcium carbonate, vitamin D and calcimimetics; however, she had mild parathyroid swelling (4.8 mm × 9.0 mm × 7.5 mm). These results suggested a severe impairment of bone material quality apart from CKD-MBD. Thus, to investigate the cause of her bone abnormality and skin sclerosis, iliac bone and femoral skin biopsies were planned on admission. Upon admission, characteristic findings of Noonan syndrome including facial feature abnormalities, short stature, and a broad/webbed neck were found. Her mental development was normal. Skin findings including edema and painful sclerosis were detected, but no redness or ulceration of the skin were observed. The patient was taking calcium carbonate (500 mg/d), calcitriol (2.5 μg/d) and cinacalcet hydrochloride (25 mg/d). Laboratory data showed normal levels of serum calcium (10.1 mg/dL) and serum phosphate (3.0 mg/dL). Other laboratory data including bone metabolism markers are shown in Table 1.

**Table 1:**
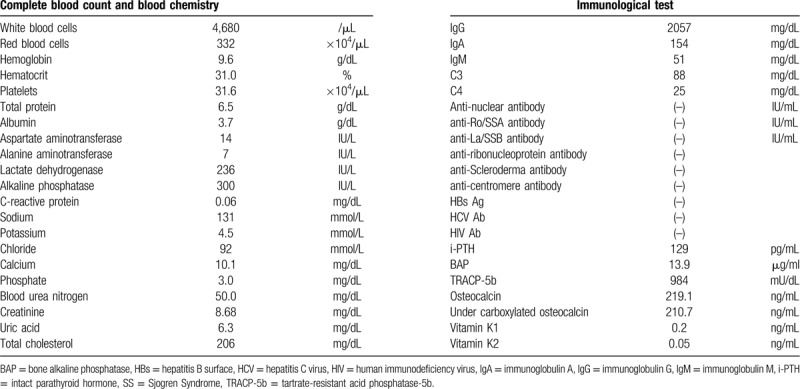
Laboratory data on admission.

**Figure 2:**
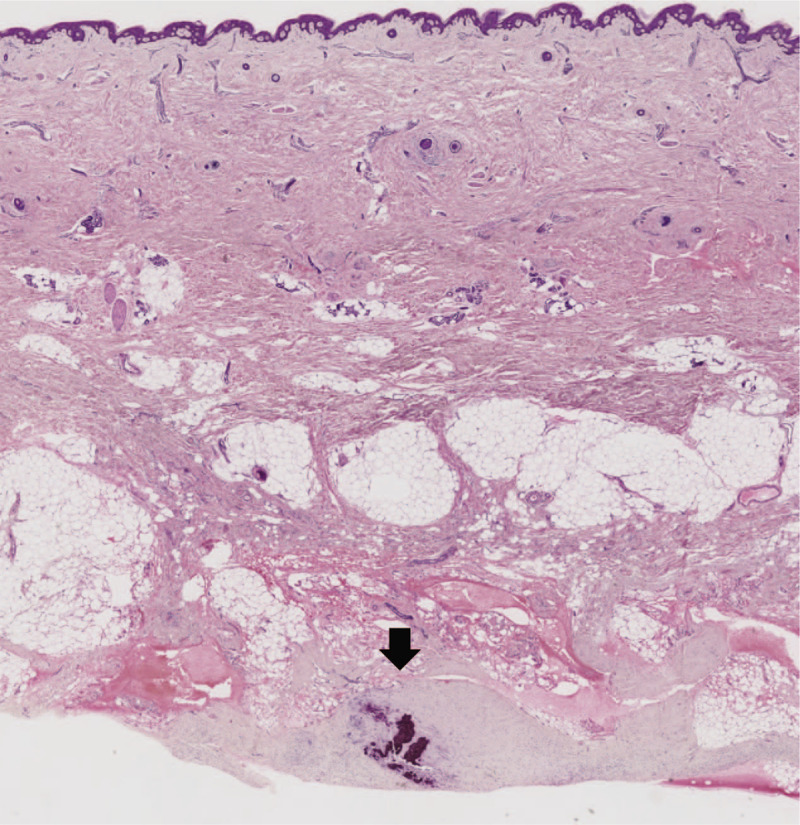
Skin biopsy findings. Skin biopsy specimens show calcification of the subcutaneous tissue.

**Figure 3:**
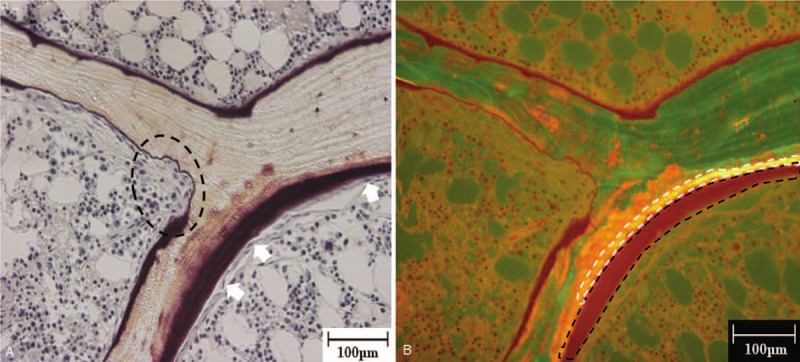
Bone biopsy findings. (A) Increased erosion (Figure 3A, black broken line) and numerous osteoclasts (Figure 3A, white arrow) on the bone surface. The volume of osteoid is greatly increased (Figure 3B, black broken line), and only 1 tetracycline-labeled band is present (Figure 3B, white broken line).

A skin biopsy specimen from the right lower extremity showed severe calcification in the subcutaneous tissue (Fig. 2, black arrow). A bone biopsy was undertaken for bone histomorphometry analysis using the tetracycline double-labeling method.^[10]^ Briefly, 14 days before the bone biopsy, tetracycline (600 mg/d) was administered for 2 days following a drug-free interval of 5 days. This course was repeated twice. Then, a biopsy of the iliac crest was performed. The bone histomorphometry analysis showed increased erosion (Fig. 3A, black broken line) and a number of osteoclasts (Fig. 3A, white arrow) on the bone surface. A large volume of osteoid (Fig. 3B, black broken line), and only 1 tetracycline-labeled band (Fig. 3B, white broken line) were observed in most areas, suggesting impairment of bone calcification processes; normal bone calcification is indicated by the presence of 2 tetracycline-labeled bands. Some mild fibrosis was observed. The results for other histomorphometric parameters are shown in Table 2. These bone biopsy findings led to a diagnosis of high bone turnover, low mineralization, and low bone volume (Table 3). The patient's bone disease could not be categorized into any type of representative bone disease classification such as osteitis fibrosa, osteomalacia, adynamic bone disease, uremic osteodystrophy or hyperparathyroidism in accordance with turnover, mineralization, and volume classifications (Table 3)^[11,12]^, but was consistent with warfarin-induced impairment of bone material quality (Table 3).^[13]^ These results led to a diagnosis of bone material quality impairment induced by warfarin. However, warfarin could not be suspended because of the increased risk of blood clots caused by the implanted mechanical aortic valve. Hence, treatment with a bisphosphonate (monthly intravenous ibandronate 1 mg), which binds to the bone mineral surface and inhibits osteoclasts, was started after diagnosis. Her skin sclerosis and clinical symptoms, such as lower extremity pain and limited range of joint motion, improved after 1 year of treatment with bisphosphonates. Furthermore, bone density was maintained and calcification in the subcutaneous tissue did not progress.

**Table 2:**
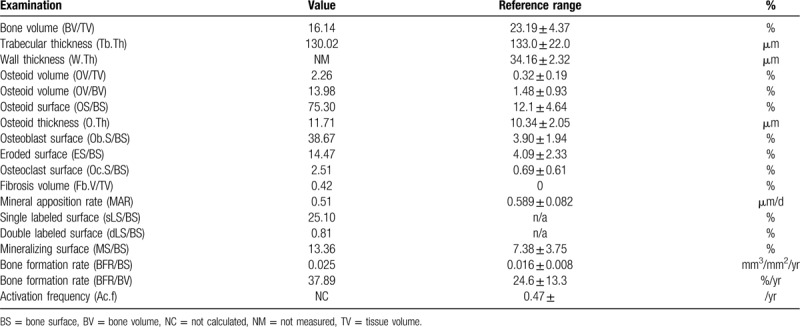
Bone histomorphometry analysis.

**Table 3:**
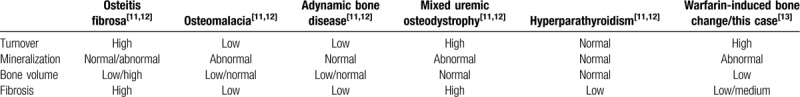
Bone disease classification by turnover, mineralization, and volume.

## 3 Discussion

We report a case of warfarin-induced impairment of bone material quality evaluated by bone histomorphometry in a patient who was undergoing hemodialysis.

Previous studies have reported that the use of warfarin is associated with an increased incidence of bone fractures in patients undergoing hemodialysis^[14]^; however, no studies have directly estimated bone quality by bone histomorphometry in patients with bone abnormalities who are taking warfarin. To our knowledge, this is the first report of warfarin-induced bone material quality impairment evaluated by bone histomorphometry in a patient undergoing hemodialysis.

In this case study, the bone histomorphometry analysis showed increases in erosion, the number of osteoclasts on the bone surface, and the osteoid volume. A significant reduction in the double-labeled surface was observed without diffuse fibrosis; this patient exhibited only one tetracycline-labeled band in most areas of bone. This bone abnormality was not representative of any type of bone disease in the turnover, mineralization, and volume classification.^[11,12]^ A previous study reported that warfarin-treated animals showed increases in erosion and the number of osteoclasts on the bone surface without fibrosis.^[13]^ Furthermore, long-term warfarin administration was shown to impair bone material quality in a rat experimental study.^[15]^ These reported effects of warfarin led us to conclude that the patient's bone disease was induced by warfarin. However, because our patient had end-stage renal disease and was undergoing hemodialysis, it was necessary to consider the effects of CKD-MBD including secondary hyperparathyroidism as a possible cause of bone disease in this case. Secondary hyperparathyroidism is characterized by an increased level of serum parathyroid hormone. The serum parathyroid hormone level is positively correlated with bone turnover in CKD-MBD.^[16]^ Additionally, an increased level of parathyroid hormone results in bone fibrosis and deficient bone mineralization.^[17]^ In this case, the parathyroid hormone level was not high, and the bone fibrosis was quite mild; hence, it was unlikely that secondary hyperparathyroidism significantly contributed to the development of her bone disease. We observed an increase in the osteoid volume and a decrease in the double tetracycline labeling surface area in this patient. These observations indicated a deficiency in bone mineralization. Although deficient bone mineralization occurs in osteomalacia^[11]^, the bone histomorphometry analysis of this patient showed no evidence of osteomalacia. Furthermore, this patient did not have any predisposing factors for osteomalacia such as vitamin D deficiency or iron overload.^[18]^

In this case, the ratio of undercarboxylated osteocalcin to carboxylated osteocalcin (undercarboxylated osteocalcin/carboxylated osteocalcin) in the serum was markedly elevated at 0.96 (normal range: <0.15–0.57). This ratio is regarded as a metabolic marker of vitamin K status in bone.^[19]^ In this patient, undercarboxylated osteocalcin was elevated by negative feedback; its conversion to carboxylated osteocalcin may have been blocked by warfarin.^[6]^ Additionally, X-ray and skin biopsy specimens of this patient showed severe calcification of the fascia, tendons and subcutaneous tissue. Such calcification can be explained by warfarin-mediated inhibition of vitamin K-dependent gla-protein which acts as a strong inhibitor of tissue calcification.^[20]^ Noonan syndrome is not characterized by poor bone material quality. Taking all these factors into consideration, we concluded that the impaired bone material quality in this case was induced by warfarin. We administered a bisphosphonate (monthly intravenous ibandronate 1 mg) to inhibit osteoclast activity and suppress bone remodeling because the high turnover of bone was considered to be one of the factors accelerating the patient's bone disease.^[21]^ Her skin sclerosis and clinical symptoms, such as lower extremity pain and limited range of joint motion, improved after 1 year of treatment with bisphosphonates. Furthermore, her bone density was maintained and calcification in the subcutaneous tissue did not progress. These results suggest that bisphosphonates may be effective at least partially in reducing warfarin-induced impairment of bone material quality in a patient undergoing maintenance hemodialysis. However, these effects of bisphosphonates need to be investigated carefully over a longer period.

Recently, warfarin has been replaced in many cases with direct-acting oral anticoagulants for several reasons including drug interactions, a lower risk of major bleeding, and possible improvement in stroke prevention rates.^[22]^ However, some concerns have been raised regarding the safety of direct-acting oral anticoagulants in patients undergoing dialysis. The development of direct-acting oral anticoagulants that can be safely used even in patients undergoing hemodialysis are yet to be developed.

In conclusion, we report a case of a patient undergoing hemodialysis who was diagnosed with warfarin-induced impairment of bone material quality. The analysis of bone histomorphometry was useful in the diagnosis of this bone disease.

## Acknowledgments

We thank Helen Jeays, BDSc AE, from Edanz Group (https://en-author-services.edanzgroup.com/) for editing a draft of this manuscript.

## Author contributions

**Conceptualization:** Satoshi Kurihara.

**Data curation:** Hiroki Ishii.

**Investigation:** Keiji Hirai, Katsunori Yanai.

**Methodology:** Satoshi Kurihara, Susumu Ookawara.

**Writing – original draft:** Hiroki Ishii.

**Writing – review & editing:** Satoshi Kurihara, Yoshiyuki Morishita.
